# High prevalence of cognitive impairment after intracerebral hemorrhage

**DOI:** 10.1371/journal.pone.0178886

**Published:** 2017-06-01

**Authors:** Mélanie Planton, Laure Saint-Aubert, Nicolas Raposo, Laura Branchu, Aicha Lyoubi, Fabrice Bonneville, Jean-François Albucher, Jean-Marc Olivot, Patrice Péran, Jérémie Pariente

**Affiliations:** 1 Department of Neurology, Toulouse University Hospital, Toulouse, France; 2 Toulouse NeuroImaging Center, Université de Toulouse, Inserm, UPS, France; 3 Department of Neurobiology, Care Sciences and Society, Center for Alzheimer Research, Division of Translational Alzheimer Neurobiology, Karolinska Institutet, Stockholm, Sweden; 4 Department of Neurology, Groupe Hospitalier Lariboisière-Fernand-Widal, Assistance Publique Hôpitaux de Paris, Paris, France; 5 Department of Neuroradiology, Toulouse University Hospital, Toulouse, France; Nathan S Kline Institute, UNITED STATES

## Abstract

**Background:**

Cognitive impairment seems to be frequent in intracerebral hemorrhage (ICH) survivors, but remains widely understudied. In this study, we investigated the frequency and patterns of vascular cognitive disorders (VCDs) in patients with cerebral amyloid angiopathy (CAA)-related and deep ICH compared to patients with mild cognitive impairment due to Alzheimer’s disease (MCI-AD) and healthy controls.

**Methods:**

We prospectively recruited 20 patients with CAA-related lobar ICH, 20 with deep ICH, 20 with MCI-AD and 17 healthy controls. Patients with cognitive decline pre-ICH were excluded from the analysis. Each participant underwent a comprehensive neuropsychological assessment and a structural brain MRI. Cognitive assessment was performed at a median delay of 4 months after the acute phase in ICH patients, and more than 6 months after the first complaint in MCI-AD patients. Cognitive profiles were compared between groups. The prevalence of VCDs in the ICH groups was estimated using the recent VASCOG criteria.

**Results:**

“Mild” and “major VCDs” were respectively observed in 87.5% and 2.5% of all ICH patients. Every patient in the CAA group had mild VCDs. No significant difference was observed in cognitive functioning between CAA-related and deep ICH patients. The most impaired process in the CAA group was naming, with a mean (±standard deviation) *z*-score of -5.2 ±5.5, followed by processing speed (-4.1±3.3), executive functioning (-2.6 ±2.5), memory (-2.4 ±3.5) and attention (-0.9 ±1.3). This cognitive pattern was different from the MCI-AD patients, but the groups were only different in gestural praxis, and by construction, in memory processes.

**Conclusions:**

VCDs are frequent after ICH. Cognitive patterns of patients with deep or CAA-related ICH did not differ, but there was impaired performance in specific domains distinct from the effects of Alzheimer’s disease.

**Clinical trial registration:**

URL: http://www.clinicaltrials.gov. Unique identifier: NCT01619709.

## Introduction

Cognitive impairment following intracerebral hemorrhage (ICH) has received far less attention than cognitive impairment following ischemic stroke. Most studies have focused on cerebral amyloid angiopathy (CAA) and brain microbleeds, but comprehensive neuropsychological assessment has rarely been conducted. However, recent evidence supports the notion that intracerebral hemorrhage and dementia are closely related, and that each increases the risk for the other. Moulin et al., in a prospective longitudinal study of 218 ICH survivors showed that 14.2% of patients developed new-onset dementia 1 year after ICH.[1] These findings were in agreement with those of Biffi et al., who found that 19% of patients developed incident dementia within 6 months of ICH.[2] Three studies have detailed cognitive functioning with several tests covering distinct cognitive processes after ICH or CAA-related syndromes.[3–5]In Xiong et al. and Case et al., studies [4, 5] both showed a significant impairment of processing speed and executive functions according to clinical norms or healthy controls. Impairment in processing speed as well as in episodic memory was highlighted by Arvanitakis et al. in 2011 in subjects with moderate-to-very severe CAA pathological abnormalities at autopsy, in comparison to none-to-minimal CAA subjects.[3]

In the present study, we aimed to exhaustively assess the neuropsychological performance of patients with CAA-related ICH, patients with deep ICH, patients with mild cognitive impairment due to AD (MCI-AD) and healthy controls (HC). Our main objective was to estimate the frequency of vascular cognitive disorders (VCDs) in ICH groups. The second aim was to compare CAA-related ICH group cognitive profile with those of the deep ICH, MCI-AD, and HC groups.

## Materials and methods

### Participants

Participants were prospectively recruited in the Neurology Department of Toulouse University Hospital (France). Consent was written and signed by all participants except for two patients with moderate-to-severe aphasia at the acute phase of hemorrhage. For these two cases, a close relative of the patient signed after having a specific information notice in agreement with the ethics committees. This study was approved by the local ethics committee and the French Agency for the Safety of Health Products (Refs A90605-58 & B111269-20) and written consent was obtained.

#### Survivors patients with ICH

The diagnosis of ICH was made during the acute phase on the basis of computed tomography or magnetic resonance imaging (MRI) scans. Patients were eligible for the study if they had a primary acute ICH (lobar or deep) in a supratentorial location, were older than 55 years, and were without pre-existing cognitive decline. Previous cognitive changes were estimated via the long version of the Informant Questionnaire on Cognitive Decline (IQCODE)[6] at admission. Patients with an IQCODE ≥ 3.4, reflecting pre-existing cognitive decline, were thus excluded. Patients with secondary ICH due to vascular malformation, cerebral venous thrombosis, brain tumor or anticoagulant use were also excluded. Eligible patients with ICH were divided into two groups by an experienced neuroradiologist (FB) depending on the location of the hemorrhage: either “deep” supratentorial or “lobar” (cortex or subcortical white matter). The modified Boston criteria[7] for CAA were applied to the patients with “lobar” ICH. As a result, only patients who met the criteria for possible or probable CAA were kept in this group.

#### Patients with typical MCI-AD[8]

This population was previously described.[9] Patients between 60 to 85 years with a memory complaint lasting more than 6 months were recruited. Each patient underwent clinical and neuropsychological tests, structural brain MRI and cerebrospinal fluid biomarker sampling. Patients with a diagnosis of MCI-AD met the following inclusion criteria[8]: Clinical Dementia Rating = 0.5; sum of the three free recalls ≤17/48 and/or sum of the three free and cued recalls ≤40/48 in the Free and Cued Selective Reminding Test (FCSRT)[10]; amyloid positivity on either a cerebrospinal fluid sample (level of phospho-tau (P-tau) ≥ 60 pg/ml and Innotest Amyloid Tau Index (IATI) ≤ 0.8) or ^18^F-florbetapir PET scan positivity on visual analysis.

#### Healthy controls

Individuals between 60 to 85 years with no memory complaint and no first-degree relatives with AD were recruited as healthy controls. They underwent clinical and neuropsychological tests and a brain MRI. Subjects with any cognitive impairment on the tests or significant white matter hyperintensities on T2-weighted MRI scan (Fazekas[11] score >2) were excluded.

Detailed information about each participant, including demographics, clinical status and risk factors, were prospectively recorded at the time of inclusion. Apolipoprotein E genotyping was obtained for patients.

### Neuropsychological assessment

Neuropsychological assessment was performed after the third months to decrease the influence of vigilance, attentional and language parameters on cognitive performance. We selected 10 neuropsychological tests covering 13 cognitive functions to comprehensively assess cognitive processes. All tests and assessment techniques are detailed in Lezak.[12] Overall cognitive status was assessed by the Mini Mental State Examination. The FCSRT and the Delayed Matched Sample tests were chosen to assess anterograde memory processes (immediate, free and cued recall, and recognition processes). The Frontal Assessment Battery was used to evaluate overall executive function. Executive processes were explored with the Trail Making Test for flexibility, the Stroop test for inhibition, phonemic and semantic verbal fluency for initiation, and the Digit Span Forward and Backward (WAIS-III) for verbal working memory. We used the raw response times (seconds) for the TMTA and Stroop naming and reading to assess information processing speed. The TMTA and Stroop naming error scores were used to assess selective attention. A picture naming test was chosen for language, and Mahieux’s test for ideational and ideomotor apraxia. Finally, depression was measured with the Montgomery and Asberg Depression Rating Scale in the ICH groups, and with the Beck inventory in the MCI-AD and control groups, with clinical cut-off scores of 6/60 and 3/39, respectively.

### Criteria for VCDs

The incidence of vascular cognitive disorders in our ICH groups was defined using the VASCOG criteria, in which VCDs are defined as either “mild” or “major”.[13] Mild VCD is defined as a documented or inferred acquired decline in one or more cognitive domains, with test performance between 1 and 2 standard deviations (SDs) below the appropriate norm. Conversely, major VCD is diagnosed when performance is 2 or more SDs below the norm, with interference in activities of daily living.

### Brain MRI

MRI images were acquired on a Philips 3T imager (Intera Achieva, Philips, Best, The Netherlands). For all patients with ICH, the hematoma was manually segmented (MP) on native-space T1 images using MRIcro software (http://www.mccauslandcenter.sc.edu/crnl/tools), and registered into Montreal Neurological Institute space, using the transformation matrix of the non-linear registration of the T1 to generate lesion maps for each ICH group. Neuroimaging markers of cerebral small vessel disease were defined according to the STandards for ReportIng Vascular changes on nEuroimaging (STRIVE) criteria,[14] for severity of white matter hyperintensities using the Fazekas scale,[11] the number of cerebral microbleeds scored with the BOMBs scale,[15] and the presence or absence of superficial cortical siderosis.

### Statistics

Clinical as well as imaging features in the two ICH groups were compared using T-tests for independent groups, or Pearson Chi-square tests when appropriate. General linear model (GLM) factorial analyses of variance (ANOVAs) were applied with Bonferroni *post hoc* comparisons, in order to compare the four clinical groups on demographic and cognitive data. The verbal memory and Stroop scores of ICH patients with mild-to-moderate aphasia at the time of the neuropsychological assessment (*n* = 2) were not taken into account in the comparative analysis. For a visual representation of cognitive results, patients’ individual cognitive test scores were converted to *z*-scores using the means and SDs of the HC group as a reference. Based on these *z*-scores, measures summarizing the different cognitive processes (means) were calculated for all patient groups. Using this method, we obtained *z*-scores representing each of the 13 functions. We also used *z*-scores to determine the VCDs prevalence in the ICH groups. We used STATISTICA software (StatSoft, Tulsa, OK, USA) to perform all statistics.

## Results

**Fig 1:**
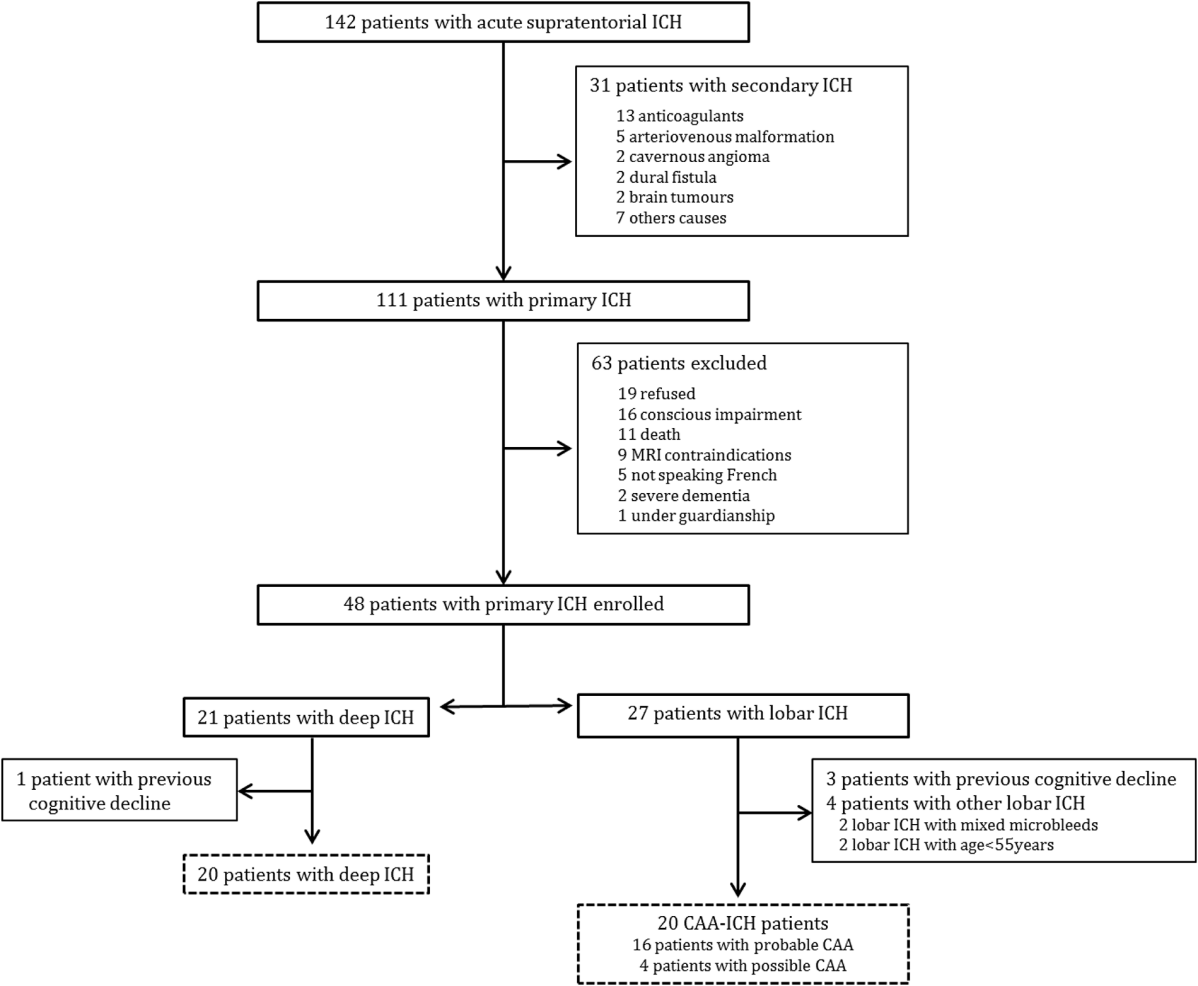
Flow chart for patients selection.

Of the 142 patients screened with acute supratentorial ICH, 40 patients met the inclusion criteria (Fig 1). Of these 40 patients, 20 had deep ICH and 20 CAA-related ICH (4 possible, 16 probable CAA with 2 with supporting pathology). Twenty patients with MCI-AD and 17 HC were also recruited. No difference was found between these 4 groups for age, education and gender. ApoE genotypes did not differ between the 3 patient groups (Table 1).

**Table 1:** Comparison between groups of demographic and clinical profiles at inclusion.

Number	Deep ICH	CAA	MCI-AD	HC	*P* value
	20	20	20	17	
Age (years), mean (SD)	65.8 (10.3)	70.7 (9.3)	71.6 (4.5)	69.9 (4.8)	.101
Gender (F/M)	7/13	13/7	9/11	10/7	.455
Education (years), mean (SD)	11.1 (4.1)	11.8 (6.2)	11.5 (2.7)	12.8 (3.3)	.694
IQCODE score, median [IQR]	3.03 [3–3.19]	3.02 [3–3.12]	-	-	.268
Disease duration (years), mean (SD)	-	-	3.5 (3.3)	-	-
Delay from ICH to MRI scan (days), median [IQR]	12 [8–20]	14 [8–23]	-	-	.150
Delay from ICH to neuropsychological assessment (days), median [IQR]	125 [117–147]	108 [98–139]	-	-	.606
Biological characteristics*					
ApoE allele ε2, No. (%)	2/20 (10)	5/17 (29.4)	3/17(17.6)	-	.776
ApoE allele ε4, No. (%)	3/20 (15)	5/17 (29.4)	8/17 (47.1)	-	
**Imaging characteristics of ICH groups**					
ICH volume (mL), median [IQR]	9.2 [0.7–21.8]	33.9 [0.8–76.6]	**-**	-	**< .001**
Right side ICH, No. (%)	13 (65)	4 (20)	**-**	-	**.003**
Total number of microbleeds, median [IQR]	3 [1–10]	5 [0–14]	-	-	.245
Patients with strictly lobar microbleeds, No. (%)	3 (15)	9 (45)	**-**	-	**.038**
Patients with superficial cortical siderosis, No. (%)	1 (5)	12 (60)	**-**	-	**< .001**
Fazekas and Schmidt score (/9), median [IQR]	6 [4–7]	8 [5–9]	-	-	.168
**Clinical characteristics of ICH groups**					
Hypertension, No. (%)	18 (90)	7 (35)	**-**	-	**.001**
Diabetes, No. (%)	4 (20)	3 (15)	-	-	.317
Dyslipidemia, No. (%)	8 (40)	2 (12.5)	**-**	-	**.014**
Obesity, No. (%)	2 (10)	2 (10)	**-**	-	>.99
Smoker, No. (%)	5 (25)	3 (15)	-	-	.157
Previous stroke, No. (%)					
Amyloid spells, No. (%)	0	2 (10)	**-**	-	.147
Antiplatelet use, No. (%)	5 (25)	4 (20)	**-**	-	.705
NIHSS, median [IQR]	5 [0–11]	5 [0–16]	**-**	-	.802
NIHSS at the follow-up visit, median [IQR]	3 [0–11]	2 [0–6]	**-**	-	.196
mRs at the follow-up visit, median [IQR]	2 [0–5]	2 [0–3]	-	-	.496

* ApoE genotyping was unavailable for 3 patients in the CAA group and 3 patients in the MCI-AD group.

ApoE, apolipoproteinE; CAA, cerebral amyloid angiopathy; MCI-AD, mild cognitive impairment due to AD; HC, healthy controls; ICH, intracerebral hemorrhage; IQR, interquartile range; NIHSS, National Institute of Health Stroke scale; mRs, modified Rankin scale.

### Clinical and imaging features in the ICH groups

The median (Inter-quartile range [IQR]) National Institutes of Health Stroke Scale scores at admission were not different (5 [0–11] for the deep group and 5 [0–16] for the CAA group, *P* = .802). Hypertension and dyslipidemia were more frequent in the deep ICH group than in the CAA group. IQCODE scores were equivalent between the two ICH groups (Table 1).

**Fig 2:**
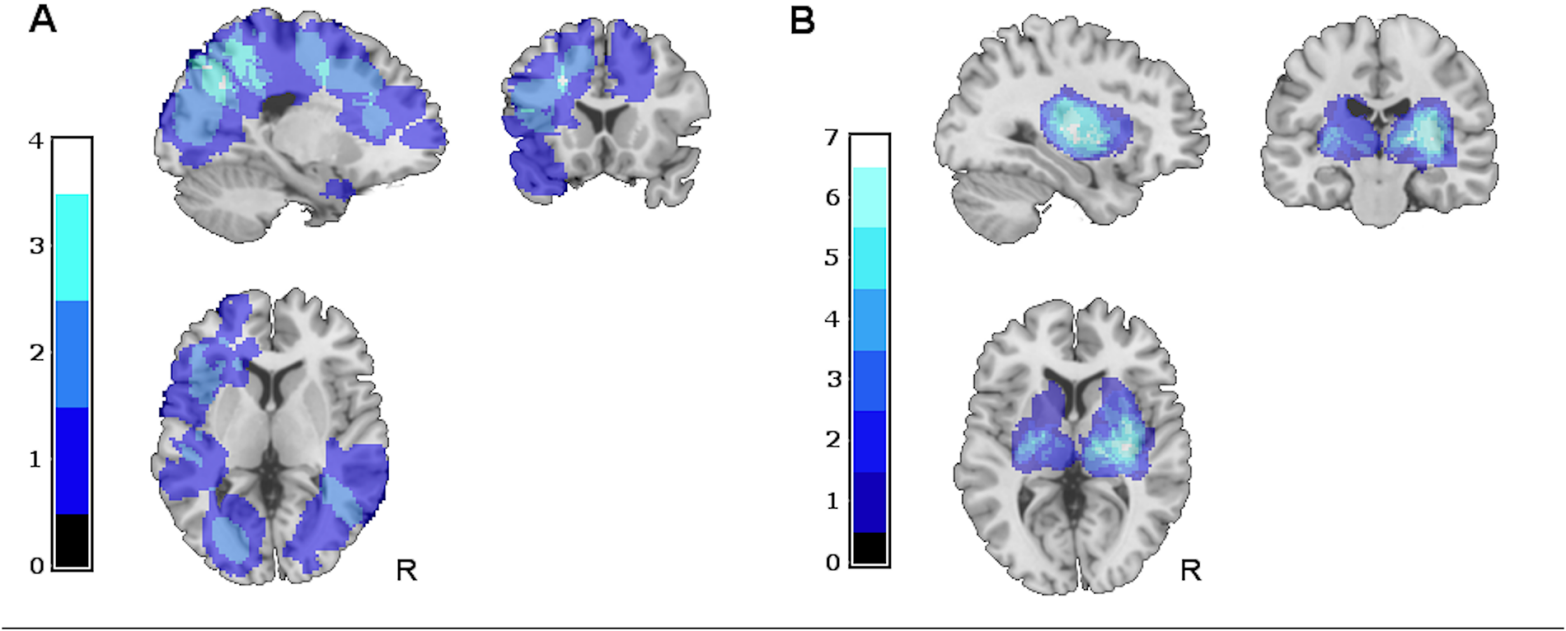
Intracerebral hemorrhage lesion maps. A. Lesion map of CAA-related ICH group. B. Lesion map of deep ICH group. Units on color scale represent number of patients with a lesion in each area.

The ICH volume was larger in the CAA group than in the deep group (33.9 mL vs. 9.2 mL respectively; *P* < .001), and more frequently located in the left hemisphere (*n* = 16 vs. *n* = 7, respectively) (Fig 2). Deep ICH caused 11 strategic lesions for cognition in the thalamus (4 left, 5 right) and the caudate nucleus (1 left, 1 right).

### Prevalence of VCDs

At a median delay of 4 months after the ICH, the neuropsychological assessment showed that 2.5% (1/40) met the criteria for major VCDs, and 87.5% (35/40) were diagnosed as having mild VCDs. Every patient in the CAA group had mild VCDs and none met the criteria for major VCDs. In the deep ICH group, 75% of patients (15/20) had mild VCDs and 5% (1/20) had major VCDs. Moreover, 8 of the 11 deep strategic ICH (72.7%) met the criteria for VCDs (1 patient had major VCDs and 7 patients had mild VCDs).

### Neuropsychological outcome

Comparison of cognitive scores 4 months after the ICH showed differences between the groups (*F*(3, 75) = 2.72, *P* < .001). For more details of raw scores and *P* values, see Table 2.

**Table 2:** Comparison of cognitive profiles among the clinical groups.

	MCI-AD	CAA	Deep ICH (D. ICH)	Healthy controls (HC)	*Bonferroni test*, *P value*					
					*MCI-AD vs CAA*	*MCI-AD vs D*. *ICH*	*CAA vs D*. *ICH*	*MCI-AD vs HC*	*CAA Vs HC*	*D*. *ICH vs HC*
**Global efficiency**										
MMSE (/30)	25.5 (2.1)	24.1 (3.5)	24.9 (5.7)	28.4 (0.7)	-	-	-	-	.022	.029
**Memory**										
*Immediate recall*	13 (2,5)	14.2 (2.5)	14.6 (1.8)	15.6 (0.7)	-	.039	-	< .001	-	-
Free recall										
FCSRT (sum of 3 free recall, /48)	11.2 (5.9)	18.8 (9.4)	19.8 (9.2)	32.2 (4.6)	-	.010	-	<.001	<.001	<.001
FCSRT (delayed free recall, /16)	4.2 (3)	7.6 (4.2)	7.2 (4.5)	12.8 (2.1)	-	-	-	<.001	.001	<.001
*Cued Recall*										
FCSRT (sum of 3 total recall, /48)	28 (12.3)	38.9 (12.4)	40.5 (9.6)	46.6 (1.9)	.032	.002	-	<.001	-	-
FCSRT (delayed total recall, /16)	10.5 (5.1)	13.8 (3.7)	13.2 (3.5)	15.8 (0.6)	-	-	-	.001	-	-
*Recognition*										
FCSRT (recognition, /48)	43.4 (7.1)	47.2 (2.1)	46.5 (3.2)	47.8 (0.8)	.036	-	-	.010	-	-
DMS 48, score set 1 (/48)	40.9 (5.9)	43.3 (6.2)	42.6 (5.5)	46.5 (2)	-	-	-	-	-	-
DMS 48, score set 2 (/48)	40.2 (7.3)	42.7 (5.5)	41.4 (5.5)	45.9 (2.2)	-	-	-	-	-	-
**Executive**										
*Overall executive*										
FAB (/18)	15.3 (2.6)	14.3 (2.7)	14.2 (4.3)	17.1 (0.8)	-	-	-	-	-	.014
*Processing speed*										
Stroop denomination, time	80.1 (22.5)	106.2 (34.1)	96.6 (32.2)	67.3 (15.3)	-	-	-	-	.002	.015
Stroop reading, time	51.5 (12.6)	67.6 (19.8)	70.1 (32.2)	43.3 (5.9)	-	-	-	-	.011	.001
TMTA, time	53.2 (22.3)	89 (49.1)	74.5 (61.5)		-	-	-	-	.006	.029
*Flexibility*										
TMT B-A time	125.7 (77.3)	181.9 (92.5)	143.8 (97.8)	61 (31.1)	-	-	-	.034	.001	.010
TMT B-A Errors	1.3 (1.5)	1.7 (1.5)	1.5 (1.9)	0.5 (0.6)	-	-	-	-	-	-
*Inhibition*										
Stroop test interference score (IS), time	105.6 (42.5)	132.9 (63.6)	166.1 (262.2)	51.6 (31.9)	-	-	-	-	-	-
Stroop test IS, non-corrected errors	2.2 (3)	3.9 (5.6)	3.4 (5)	0.5 (0.7)	-	-	-	-	-	-
*Initiation*										
Verbal fluency (P)	19.9 (8.2)	11.8 (7.8)	13 (7.3)	22.6 (6.1)	-	-	-	-	.001	.002
Verbal fluency (Animals)	21.8 (7.7)	19.4 (5.8)	20.7 (11)	31.8 (7.4)	-	-	-	.001	.001	.001
*Verbal working memory*										
Digit Span forward	5.7 (1.4)	4.6 (1)	5.1 (1.1)	5.4 (1)	-	-	-	-	-	-
Digit Span backward	4.1 (1.3)	3.4 (0.9)	3.8 (1.2)	4.6 (0.9)	-	-	-	-	.027	-
**Attention**										
*Selective attention*										
TMTA, error	0 (0)	0.4 (0.5)	0.5 (0.9)	0.1 (0.2)	-	.021	-	-	-	.046
Stroop denomination, error	0.5 (0.8)	0.5 (0.6)	0.7 (1.1)	0	-	-	-	-	-	-
**Language**										
*Naming (/80)*	78 (4)	73.1 (6.3)	73.9 (9.2)	79.4 (1.2)	-	-	-	-	.023	-
**Praxis**										
*Gestural praxis (/23)*	20.5 (5)	23 (0)	22.5 (1.8)	23 (0)	.012	-	-	.012	-	-

Results are expressed as means (SD).

CAA, cerebral amyloid angiopathy; HC, healthy controls; ICH, intracerebral hemorrhage; IQR, interquartile range; MCI-AD, mild cognitive impairment due to AD; MMSE, Mini Mental State Examination; FCSRT, Free and Cued Selective Reminding Test; FAB, Frontal Assessment Battery test; TMT, Trail Making Test.

#### CAA versus deep ICH

**Fig 3:**
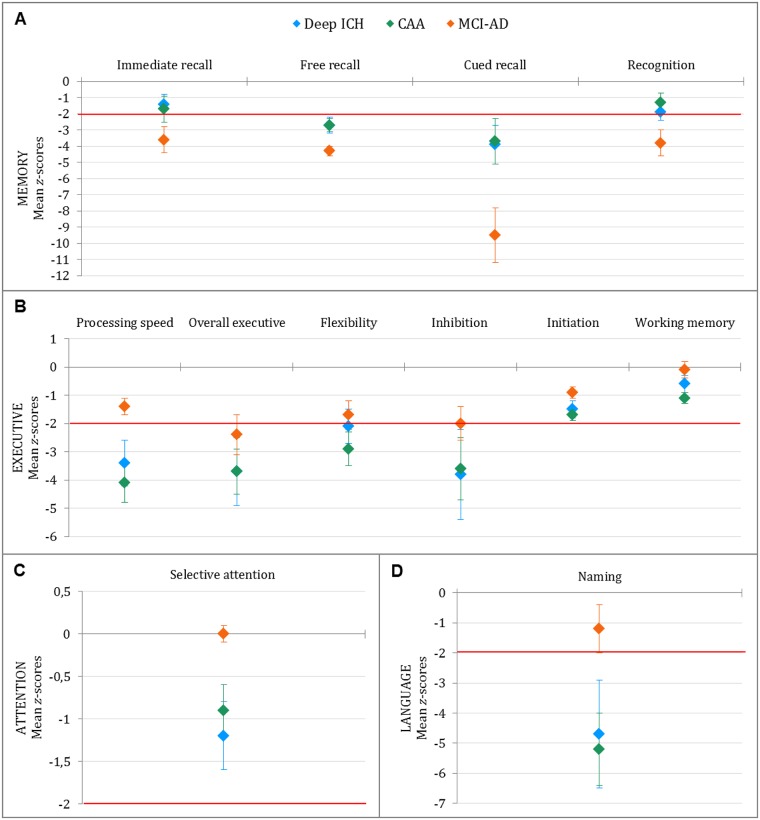
Comparison among patient groups in cognitive profiles expressed as *z*-scores. The diamond symbols represent the mean *z*-score value for the group, and error bars represent standard errors. The red line represents the threshold of 2 standard deviations from the mean performance of healthy controls.

No difference was observed in the 13 cognitive functions between the two groups (Fig 3).

#### CAA versus MCI-AD

Patients with CAA performed better than MCI-AD patients on gestural praxis and by construction on cued recall and recognition memory processes (Fig 3A).

#### Deep ICH versus MCI-AD

Patients with deep ICH scored worse than patients with MCI-AD on selective attention processes (Fig 3C), but had higher memory scores on immediate, free and cued recall (Fig 3A).

#### Patients versus controls

Compared with HC, patients with deep ICH scored worse on 7 of the 13 cognitive processes, as did the MCI-AD group. The CAA group performed worse on 5 of them. For the latter group, naming was the most impaired process (mean *z*-score: -5.2 ±5.5), followed by processing speed (-4.1 ±3.3), executive functioning (-2.6 ±2.5), memory (-2.4 ±3.5) and attention (-0.9 ±1.3).

Depression was reported in 40% of CAA patients, in contrast to the other clinical groups, who all showed a prevalence below 30% (15% of deep ICH patients, 25% of MCI-AD patients, and 17.6% of HC). However no significant difference was found between groups (Pearson chi^2^ = 3.5, *P* = .321).

## Discussion

Our results suggest that, after a median delay of 4 months post-ICH: i) mild VCDs were observed in 87.5% of all ICH patients; ii) the most impaired cognitive process in the CAA group with a mean performance more than 2 SDs below the norm of controls was naming followed by processing speed, executive functioning and memory; iii) there was no difference in cognitive patterns after either a CAA-related or a deep ICH; iv) cognitive dysfunctions in ICH patients were different from those in MCI-AD patients.

Experts on VASCOG[13] committee introduced diagnostic thresholds, groups of cognitive disorders, and clinical and neuroimaging criteria to establish the vascular etiology of VCDs, providing a major update for the diagnosis of vascular cognitive impairment. According to these criteria, 2.5% of our ICH patients met the criteria for major VCDs, and 87.5% were diagnosed as having mild VCDs (100% in the CAA group, 75% in the deep ICH group). Another way to assess cognitive impairment is to use clinical norms of the tests performed. With this method, we showed subtle difference in the prevalence of mild VCDs and no change for major VCDs. For the CAA-related ICH patients, the prevalence of mild VCDs was estimated to 95% and was of 70% in the deep ICH group. The prevalence of dementia was lower than the rates reported by Moulin et al. [1] and Biffi et al., [2] in prospective studies of ICH survivors without pre-existing dementia before ICH. Moulin et al., showed that the risk of dementia was twice as high in patients with lobar ICH than in patients with non-lobar ICH. We can assume that a subsequent cognitive assessment follow-up of ICH patients would increase the rate of dementia, especially in the CAA group. Conversely, the prevalence of mild VCDs was slightly higher than in the ischemic stroke population.[16,17] The cutoff of -1 SD or the 16th percentile for considering a performance as mildly altered, as defined by the DSM-5 and therefore by the VASCOG community, can lead to false-positives.[18] This issue must be kept in mind for future studies needed on larger populations of cerebrovascular patients, in order to assess the sensitivity and specificity of each of these criteria.

The absence of any significant difference in neuropsychological data between patients with CAA-related ICH and deep ICH was explained by the presence of multi-domain cognitive impairment in both groups. These findings could also be influenced by a lower statistical power. In the deep ICH group, the naming process was the most severely impaired compared to the control group, followed by processing speed, memory functions—specifically cued and free recall processes—and executive functions. This neuropsychological profile is observed in patients with a deep strategic ICH location for cognition.[19, 20] In our study, 72.7% of patients with a deep strategic lesion met the criteria for VCDs. The functional involvement of the thalamus in lexical-semantic functions and in word-finding[19] and for anterograde memory[21] is now widely accepted. Executive disorders following caudate, thalamic or putaminal hemorrhage have also been extensively reported, consistent with our results, where cognitive control depends on interactions between the prefrontal cortex and basal ganglia.[20, 22] Cognitive disorders in CAA patients were also frequent, in numerous domains. Patients exhibited significant deficits in language, processing speed and executive and memory functions compared to the control group, but were not different on attention and praxis domains. We showed that naming was the most impaired process, as in the deep group, followed by executive and processing speed. This pattern of “frontal” cognitive dysfunction was already highlighted in the CAA population, by Xiong et al.[5] in patients with a history of intracerebral hemorrhage, and by Case et al.[4] 90 days after symptomatic ICH in CAA patients included with ICH presentation or CAA-related-syndrome. The findings have confirmed those of recent studies in small vessel disease populations including CAA [23,24] and in CADASIL patients [25] where an association was found between global efficiency and performance on executive and/or processing speed. This commonly appears after stroke. It is important to note that the cognitive profile described in our study may also be influenced by the presence of hemorrhagic lesions in the frontal cortex. Interestingly, cognitive disorders were more severe in our study, in comparison with Xiong et al. and Case et al.,[4, 5] although the CAA patients were in the same age range. It is possible that the absence of an MRI scan controlling for possible lesions in healthy controls in the study of Xiong et al. influenced the results, thus underestimating cognitive deficits in CAA patients. Furthermore, the use of cognitive composite scores in both studies with few variables in each cognitive domain may explain the differences with our *z*-scores.

CAA patients had a specific pattern of dysfunction in comparison with the MCI-AD group. While the average alteration in MCI-AD was prominent in memory function, CAA patients were more dysexecutive and had more deficits in attentional and naming scores. Case et al. already demonstrated lower memory composite scores in AD patients compared to CAA patients, without a difference in executive and processing speeds between the groups.[4] It should be noted that the worse performance found in the MCI-AD group with regard to the other groups was obtained by construction, as MCI-AD patients were recruited based on their poor memory performance. However, memory dysfunction appeared not to be specific to MCI-AD patients. Free and cued memory mean *z-*scores for the CAA group were more than 2 SDs below the norm for the HC. This highlights the retrieval and storage memory dysfunction in the CAA group.

An important question remains: What are the mechanisms underlying the cognitive disorders in the two ICH groups? In the deep ICH group, the strategic location of the lesions is a clear factor in the observed cognitive impairment. As for the CAA group, the lesions affected various cortical regions, but with little overlap across patients. In contrast, the cognitive profile in CAA patients was homogeneous. One explanation for the mechanisms underlying such disorders may be partly related to neurodegenerative co-morbidity. Indeed, epidemiological and clinical-pathological findings indicate considerable overlap between amyloid vascular lesions and AD pathology. In the present study, we cannot definitively conclude on the underlying mechanisms of the cognitive impairment observed, particularly since the impact of markers of small vessel disease on cognition was not controlled in the 3 patient groups.

There are methodologic limitations to our study that should be discussed. The first issue is related to the sample selection and its relatively small size. This study was conducted on selected ICH patients; therefore, the results reported may not be representative of the entire ICH population. Our ICH cohort was hospital-based, and patients recruited in a neurological stroke unit are more likely to be younger and independent. This could explain the low rate of mortality in our study. In addition, we chose to include patients with MRI data scans only, which excluded patients with severe neurological conditions and consequently could explain the low rate of dementia compared to prior studies. Second, 4 of the 20 patients had “possible CAA” with the modified Boston criteria. This has a rather poor accuracy in detecting CAA and thus underestimated the severity of major VCDs. Third, although we used the long version of the IQCODE questionnaire to exclude patients with pre-ICH cognitive decline, we could not exclude that mild cognitive changes were already present and thus influenced the cognitive profile post-ICH. Nevertheless, the neuropsychological assessment proposed in this study was comprehensive and we did not use cognitive composite scores that may disguise cognitive processes. Future longitudinal studies on cognitive and imaging data should further clarify the changing nature of mild and major VCDs in the ICH population. Cognitive tests assessing gnosis functions should be added to the neuropsychological examination.
